# Development of an electronic health records datamart to support clinical and population health research

**DOI:** 10.1017/cts.2020.499

**Published:** 2020-06-23

**Authors:** Jillian H. Hurst, Yaxing Liu, Pamela J. Maxson, Sallie R. Permar, L. Ebony Boulware, Benjamin A. Goldstein

**Affiliations:** 1 Duke Children’s Health and Discovery Institute, Department of Pediatrics, Duke University School of Medicine, Durham, NC, USA; 2 Duke Clinical and Translational Science Institute, Duke University School of Medicine, Durham, NC, USA; 3 Duke Health Technology Solutions, Durham, NC, USA; 4 Duke Center for Community and Population Health Improvement, Duke University School of Medicine, Durham, NC, USA; 5 Department of Pediatrics, Division of Infectious Diseases, Duke University School of Medicine, Durham, NC, USA; 6 Department of Medicine, Division of General Internal Medicine, Duke University School of Medicine, Durham, NC, USA; 7 Department of Biostatistics and Bioinformatics, Duke University School of Medicine, Durham, NC, USA

**Keywords:** Electronic health records, PCORnet, common data model

## Abstract

**Introduction:**

Electronic health record (EHR) data have emerged as an important resource for population health and clinical research. There have been significant efforts to leverage EHR data for research; however, given data security concerns and the complexity of the data, EHR data are frequently difficult to access and use for clinical studies. We describe the development of a Clinical Research Datamart (CRDM) that was developed to provide well-curated and easily accessible EHR data to Duke University investigators.

**Methods:**

The CRDM was designed to (1) contain most of the patient-level data elements needed for research studies; (2) be directly accessible by individuals conducting statistical analyses (including Biostatistics, Epidemiology, and Research Design (BERD) core members); (3) be queried via a code-based system to promote reproducibility and consistency across studies; and (4) utilize a secure protected analytic workspace in which sensitive EHR data can be stored and analyzed. The CRDM utilizes data transformed for the PCORnet data network, and was augmented with additional data tables containing site-specific data elements to provide additional contextual information.

**Results:**

We provide descriptions of ideal use cases and discuss dissemination and evaluation methods, including future work to expand the user base and track the use and impact of this data resource.

**Conclusions:**

The CRDM utilizes resources developed as part of the Clinical and Translational Science Awards (CTSAs) program and could be replicated by other institutions with CTSAs.

## Introduction

Electronic health records (EHRs) systems have become a crucial part of healthcare provision. Healthcare providers, health systems, and payors have made substantial commitments to accurately capture patient information and to use EHR interfaces to improve care provision. EHRs also represent an important data resource as they can provide cross-sectional and longitudinal data on large “cohorts” of up to millions of individuals. Consequently, EHR data are being organized and leveraged to inform our understanding of disease progression, outcomes analyses, epidemiology, quality improvement, and comparative effectiveness research (CER) [1]. Moreover, the application of methods derived from statistics, computer science, data engineering, and informatics has resulted in a number of high-impact findings and methodologies that have the potential to transform clinical research, epidemiology, and population health sciences [2–8].

While EHR systems represent an important research data source, these data are highly complex and can be difficult to access. Typically, EHR data are stored in an enterprise data warehouse (EDW) along with a number of other data sources such as billing and claims data, laboratory tracking systems, and scheduling data that underlie health system operations. These data warehouses require significant expertise and time to navigate, and access is typically restricted to a small number of individuals to manage privacy and legal concerns associated with access to large amounts of protected health information (PHI). One way to make EHR data more accessible and actionable for research purposes is to organize it into smaller relational databases, referred to as datamarts. These datamarts are typically organized under Common Data Models (CDMs). CDMs, such as those used by the National Patient-Centered Clinical Research Network (PCORnet) and/or the Observational Medical Outcomes Partnership (OMOP), comprise a set of rules for how to turn raw EHR data into simpler data models [9]. These efforts have stimulated a significant number of retrospective analyses and innovative multicenter clinical trials [10,11].

### Objective

Due to substantial access barriers and the difficulties inherent in accessing and utilizing raw EHR data for research purposes at our institution, we sought to develop a Duke University Health System (DUHS)-specific datamart to provide well-curated and easily accessible EHR data to investigators within Duke University. In constructing a user-facing EHR data structure, our goals were to create a data platform that

- contains most data elements needed for clinical research studies;
- is directly accessible by individuals conducting statistical analyses (such as the Biostatistics, Epidemiology, and Research Design (BERD) core members or clinical scientists);
- is accessed via a code-based system to promote reproducibility and consistency across studies; and
- Utilizes a secure protected analytic workspace in which sensitive data can be stored and analyzed.

Later, we describe the development of the Duke Clinical Research Datamart (CRDM), which contains curated and well-characterized health data that can be accessed and analyzed using standardized methods that preserve data provenance and reproducibility. The CRDM builds off of our institution’s instance of PCORnet; as such, the CRDM is designed to support retrospective analyses of clinical data, provide reliable data with which to build study cohorts and registries, and contribute to the development of population-level health studies.

## Materials and Methods

DUHS utilizes the Epic EHR data platform, with all health system data stored in an EDW, including data related to patient demographics, diagnosis and procedure codes, laboratory orders and results, medication orders and fulfillments, vital signs, encounter location information, provider notes, and other detailed clinical data. The EDW contains records for encounters at three Duke-affiliated hospitals and over 300 outpatient clinics, and are refreshed nightly. The Duke CRDM utilizes data within the EDW as described below.

### Description of Data Structure

**Fig. 1.**
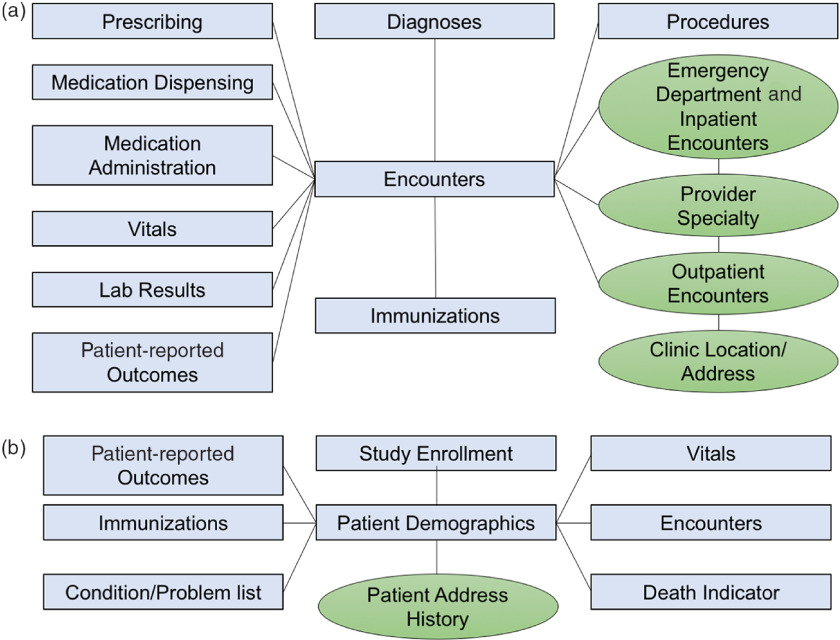
Relational table structure of the Duke Clinical Research Datamart. (A) Encounter-linked data tables. (B) Patient-linked data tables. PCORnet-derived tables are in blue/squares; data sidecars are shown in green/circles.

The CRDM utilizes data that have been transformed and cleaned for use as part of the PCORnet data network. Three hundred and forty-eight health systems from across the country participate in PCORnet, which relies on a CDM created by the Patient-Centered Outcomes Research Institute (PCORI) and licensed under Creative Commons [11], making the model freely accessible and shareable across sites. The most recent version of the PCORnet CDM (released in September 2019 [12]) defines and standardizes mapping of data elements within 22 domains. Notably, the CDM protects patient identity by assigning each patient an arbitrary identifier (Patient_ID) rather than a medical record number and utilizes standard terminologies to ensure consistent definitions within each domain. The CRDM utilizes the PCORnet domains shown in blue in Fig. 1. Based on Duke’s participation in the PCORnet network, the base PCORnet CDM is updated on a quarterly basis. Each domain in the CRDM is housed in a separate table (Fig. 1) in the form of a Structured Query Language (SQL) relational database. Each table contains at least one element (including Patient_ID, Encounter_ID, or Provider_ID) that allows data elements from each table to be linked together to provide specific sets of elements for a given encounter or patient.

### Additional Data Elements Not Included in the PCORnet CDM

The PCORnet CDM contains most of the important basic data elements for most clinical research studies; however, we found there were additional contextualizing factors that clinical researchers at our institution desired, such as address history, clinic location, and provider information. Therefore, we built tables for additional data elements (referred to as “data sidecars,” which are linked to and support the primary data tables). In order to identify data sidecars to be included in the CRDM, we met with clinicians and investigators across disciplines and identified additional data needs through testing of different use cases. Data sidecars that have been included in the CRDM are shown in green in Fig. 1. These data elements are refreshed quarterly in conjunction with the general Duke PCORnet refresh so that data are comparable and linkable across both PCORnet and sidecar domains.

### CRDM Use Cases

The CRDM was designed to facilitate work within population health, comparative effectiveness research/pharmacoepidemiology, predictive modeling, and the development of patient registries, which are discussed below. Example projects are shown in Table 1.

**Table 1.** Clinical research datamart use case examples

Use case domains	Example projects
Population Health	Multifactorial analysis of pediatric asthma outcomes Impact of neighborhood gentrification and eviction on rates of chronic diseases Evaluating healthcare utilization patterns of patients with nonalcoholic fatty liver disease
Comparative Effectiveness Research and Pharmacoepidemiology	Evaluation of opioid prescriptions in the postsurgical setting Use and effectiveness of biologic therapies for patients with asthma and chronic urticaria Lipoprotein A (Lp(a)) testing in patients with cardiovascular disease risk.
Predictive Modeling	Evaluation of a readmission risk score in hospitalized patients Prediction of healthcare utilization in patients with type 2 diabetes
Patient Registries	Health outcomes and healthcare utilization patterns in pediatric patients with epilepsy Growth trajectories in pediatric patients who are obese

#### Population health

Population health is defined as “the health outcomes of a group of individuals, including the distribution of such outcomes within a group [13],” including health outcomes, patterns of health determinants, and the factors that link the two, including policies and interventions. EHR data provide longitudinal information about large groups of individuals who receive care within a health system, making it possible to define the incidence and prevalence of different diseases, disease outcomes, and the associated factors and/or exposures associated with a given disease and related outcomes. The incorporation of time-resolved addresses allows for geospatial mapping of patients to link in external data on the built environment. As an example, our group is currently using the CRDM to evaluate comorbidities, patterns of healthcare utilization, and outcomes for children with asthma who live in Durham County, North Carolina.

#### Comparative effectiveness research and pharmacoepidemiology

CER using real world data (RWD), such as data derived from EHRs, is emerging as an important tool to evaluate the impact of different therapeutic modalities beyond the effects that can be gleaned from randomized controlled trials (RCTs). RWD-based CER allows evaluation of a broader, more heterogeneous group of patients than would have been included in given therapy’s original RCT and can provide information on the impact of comorbidities, drug–drug interactions, and the acceptability and feasibility in different clinical settings. The CRDM is currently being used to evaluate the impact of opioid prescriptions on children in a postsurgical setting. Rather than randomizing a select group of patients to a specific dosing strategy after a predefined set of procedures, the data from the CRDM allow investigators to evaluate practice patterns in a large group of patients for an extended period of time. Furthermore, the CRDM provides information about provider practice patterns in a nontrial setting, such that the data are reflective of normal practice patterns rather than of selected providers who are consciously collecting data, which may introduce practice bias. The CRDM is also being used to evaluate use of monoclonal antibodies for the treatment of severe asthma in children and adults. In the original trials, these agents were tested in study populations that were predominately white, were not large enough to evaluate the impact of common comorbidities on efficacy, and did not provide clear guidelines to help identify patients most likely to benefit from a given mAb therapeutic. The data in the CRDM provide information about the patients who are and, maybe more importantly, are not receiving mAbs, thereby providing insights into the populations that may derive the greatest benefit from these therapies.

#### Predictive modeling

Given the large number of individuals covered and the variety of features and outcomes captured, there is significant interest in using EHR data for predictive modeling. Potential applications that could utilize EHR data include clinical decision support, readmission prevention, adverse event avoidance, and chronic disease management. Currently, our group is using the CRDM to develop a risk score for hospital readmissions within 30 days of an initial hospitalization. The detailed, longitudinal data provided by the CRDM allows for rapid evaluation of risk scores using data captured by the EHR. In addition, because the patient population inclusion and exclusion criteria can be readily revised, the utility of a predictive model can be evaluated across a variety of patient groups.

#### Patient registries

Patient registries, or regularly updated lists of patients who have a particular condition or meet specific clinical criteria, are powerful tools for longitudinal evaluation of health outcomes and utilization patterns and can also be used to assess feasibility of recruiting different groups of patients for prospective trials. Once the cohorts are defined and the necessary code has been generated to assemble the dataset, the code can be rerun with each refresh of the datamart in order to retrieve updated patient information. We are currently building patient registries for pediatric patients with epilepsy and obesity, among other conditions.

### Limitations of the CRDM

The development of the CRDM is an ongoing process that is largely defined by investigator and stakeholder identified use cases and needs. There are a few key weaknesses of the CRDM:

- Detailed in-hospital data: In order to simplify the data tables, the CRDM does not have time-resolved vital signs or details on transfers within the hospital.
- Billing data: We do not currently store any billing or claims data based on patient encounters.
- Unstructured data: The CRDM only has structured data and does not contain any clinical notes or imaging data.
- Real-time patient tracking: Because the CRDM is designed to be refreshed quarterly, it cannot be used for real-time tracking of patients. It is instead suited for retrospective assessments of patient cohorts or identification of patient groups who may be eligible for recruitment into a particular clinical trial.

### User Base

The CRDM’s primary user base consists of statisticians and data scientists who work directly with clinical researchers. Like many institutions, we have a browser-based data access layer for clinicians to query EHR data [14]. While these tools are useful for initial investigational work, they do not facilitate reproducible data queries. There was an expressed desire from analytic teams to be able to directly query and access EHR data. Structure Query Language (SQL) code is used to query the CRDM and database queries can be executed using different programming languages, including R, SAS, and Python. The use of SQL code to query the database ensures that the process by which data sets are created can be easily tracked, evaluated, and replicated, and SQL code can be shared between users, making it possible to easily recreate aspects of different data sets. Training modules are being developed by which clinical researchers with a data science background could be trained in SQL to directly query the CRDM.

### Promoting Consistency in Cohort Development and Data Retrieval

One of the challenges with EHR data is that it must be processed in order to be used for research. For example, there is no field within the EHR that indicates whether a patient has diabetes; instead, one has to construct a “computable phenoytpe” [15] via a combination of diagnosis codes, laboratory test results, and/or medications. While there is arguably no “best” phenotype definition, identification and dissemination of such phenotypes can promote consistency across research studies. We are developing a code-based phenotype bank that users can directly apply when constructing their data sets. Sharing these and other best practices in a code-based manner ensures greater consistency across studies.

All registered CRDM users have access to a Gitlab page that provides a data dictionary, instructions for getting started, a list of best practices, and sets of SQL commands and associated cohort definitions that can be used for practice queries. The Gitlab also serves as a central repository for each project that utilizes the CRDM. This code repository, which will grow with continued use, serves two functions: (1) to document the methods used to create analytic datasets and (2) to provide a mechanism through which users and administrators can share known issues, best practices, and methodologies for creating datasets. As the code repository grows, access to this code base will improve the efficiency with which users are able to assemble datasets and enhance the consistency between studies.

### CRDM Access Environment

A major motivation behind the CRDM was to allow the DUHS research community the opportunity to access EHR data in a safe, consistent, and reproducible manner. As such, the CRDM is housed within a server that can only be queried while a user is logged into Duke’s Protected Analytics Computing Environment (PACE), a secure virtual network space where approved users can analyze and work with PHI and protected identifiable information (PII).

### CRDM Regulatory and Access Governance

The development of the CRDM was approved by Duke University School of Medicine’s IRB under a database and specimens repository research summary. The IRB approved the incorporation of data held within Duke’s EDW as well as quarterly updates of any data held within the CRDM. The IRB allows limited testing to ensure CRDM functionality, but does not cover the use of the data for research or quality improvement purposes. Any investigator who wishes to access and use data derived from the CRDM must file a separate IRB protocol that indicates that they will be utilizing the CRDM and specifies which data elements will be used and to what purposes.

Requests for access to the CRDM are initiated by filing out a CRDM Project Intake form. Users provide information about regulatory approvals, the data elements that will be needed for the project, funding source, and the names of the individuals who will be querying the database. After ensuring that the IRB covers the stated use and personnel, a request is made to Duke Health Technology Solutions (DHTS) to grant CRDM access. The CRDM user is also required to send a short narrative of the use case and to sign an agreement governing programmatic access to enterprise data resources that acknowledges all institutional policies regulating use of the data.

## Dissemination and Evaluation Methods

The design of the CRDM was driven by interactions with stakeholder groups, including cores of Duke’s Clinical and Translational Sciences Institute (CTSI); representatives from the Departments of Medicine, Pediatrics, and Surgery, which together represent the three largest clinical departments in Duke University School of Medicine; and an EHR datamart working group, which included representatives from CTSI cores, academic departments, and relevant institutes and centers from across Duke University. The working group met monthly in order to identify priorities for datamart design, evaluate development strategies, and discuss best practices for governance and access. CRDM design was also informed by experiences garnered from pilot projects spearheaded by members of the working group.

Members of the Duke CTSI BERD core and datamart working group also served as the first set of CRDM test users. The working group developed a statistical analysis plan that defined a clinical cohort and dataset. Test users were tasked with creating the requested dataset using the CRDM and recorded the length of time that was required, any necessary skills required for assembling the dataset, and any technical issues that were encountered during use of the CRDM. We found that users needed to be familiar with SQL commands, table joins, and to have some degree of familiarity with the PCORnet CDM in order to successfully assemble datasets.

Evaluation of the CRDM is ongoing, and the data model, governance, and access environment will be modified in order to adjust to user and institutional needs. We are currently collecting information about the departments and divisions using the CRDM, the number of manuscripts referencing/using the CRDM, grants funded that included CRDM data, and the satisfaction of the user base. The CRDM Project Intake form currently serves as our primary data collection instrument, and requires that users indicate which data elements are needed for their project and any elements that are required for the project, but that are not currently available. We will also survey CRDM users at regular intervals to determine if they have generated research products or grant applications related to the datasets that they derived from the CRDM. The datamart working group has been converted to an internal advisory board, which holds quarterly meetings to evaluate datamart use metrics (described above) and to advise on changes and upgrades to improve the usability and accessibility of the CRDM.

## Discussion

We developed a CRDM that utilizes the existing PCORnet CDM and infrastructure in order to create a regularly refreshed and directly accessible source of EHRs data for research and quality improvement purposes. The overarching goal of the CRDM is to serve the growing need for EHRs-derived datasets to be used in population health studies, comparative effectiveness research, and quality improvement, and to provide an efficient and reproducible mechanism for constructing EHR datasets. Furthermore, by expanding EHR data access, we hope to increase the pool of investigators, statisticians, analysts, and trainees working with these data in order to answer questions related to health, patient well-being, and healthcare delivery.

The CRDM utilizes resources that have been developed as part of the Clinical and Translational Science Awards (CTSA) program and could be replicated by other institutions with CTSAs. Academic medical centers that participate in PCORnet have the clinical research data network infrastructure that is a critical component of the CRDM, as the data have already been cleaned and curated and therefore adhere to a widely used data model. In order to use these data to create a CRDM-like structure, each center would need to work with their information technology departments to identify a platform where these data could be stored that would be both HIPAA-compliant and directly accessible to approved investigators and research staff. DUHS has developed the PACE, which is a secure virtual computing environment for this purpose. Similar environments are becoming available at academic medical centers to facilitate the use of patient data while providing data security. The data sidecars have been designed to provide site-specific contextual details while harmonizing with the PCORnet CDM, such that the sidecars directly link to and can be refreshed with the rest of the PCORnet instance. Other centers could define their own sidecars and would also be able to replicate the sidecars from the Duke CRDM by sourcing data from similar elements in their own data warehouses. In addition, the CRDM user base primarily consists of CTSA cores, including the BERD core and informatics core. These cores are also present at other CTSAs and can be leveraged to build data resources. The CRDM model can therefore be replicated at most large academic medical centers.

Future work with the CRDM will focus on training of an expanded user base, dissemination of best practices, and evaluation of user metrics. We are currently developing presentations and online learning modules to provide information about the CRDM that will help potential users to identify the best data resource for their research questions and quality improvement projects. We are also evaluating how best to train BERD core members and other statistics and analytics trained groups, including clinician researchers, in the use of the CRDM, including SQL coding and best practices for assembling patient cohorts and analytic datasets. As described above, we are beginning to track the use of the datamart, including any research products and grant applications supported by its use.

Expanding the pool of investigators utilizing EHR data to answer research questions is one of the primary goals of the development of the CRDM. While providers spend a significant amount of time inputting data into EHR systems, many have not had the opportunity to utilize these data to answer clinical research questions. Moreover, the analysis of EHR data can be complex, and it is important to understand how data capture mechanisms should influence the interpretation of results. In order to help familiarize investigators with the use of EHR data for research purposes, we are working with Duke’s CTSI and the Clinical Research Training Program to develop a set of didactic courses that will serve as an introduction to research uses of EHR data. Such training sessions could potentially be shared across CTSA sites that are using similar data models. In addition, we are actively working to pair investigators from different clinical specialties with informaticians, biostatisticians, and researchers who have previously used EHR data in order to address specialty-specific topics. These pairings will serve to enhance the skill sets of all investigators by serving as a conduit for sharing knowledge about the use of health data and clinical questions that can be investigated using this new data resource.

## Conclusion

The CRDM was developed with the input of multiple stakeholder groups from across Duke University School of Medicine and leverages existing data resources, including the DUHS electronic data warehouse, the PCORnet CDM, and the data that have been cleaned and standardized for incorporation into the CRDM for use in the Duke PCORnet instance. Moreover, the CRDM is available to support CTSA cores including Bioinformatics and BERD, among others. Given that these resources are available at other Clinical and Translational Sciences Award sites, we believe that the development of the CRDM could be readily replicated at other academic medical centers.
